# Hydrothermal carbonization (HTC) of dairy waste: effect of temperature and initial acidity on the composition and quality of solid and liquid products

**DOI:** 10.12688/openreseurope.14863.2

**Published:** 2022-07-12

**Authors:** Nidal Khalaf, Wenxuan Shi, Owen Fenton, Witold Kwapinski, J.J. Leahy

**Affiliations:** 1 Chemical and Environmental Science Department, University of Limerick, Limerick, Limerick, V94 T9PX, Ireland; 2 Teagasc, Environmental Research Centre, Johnstown Castle, Co, Wexford, Y35 TC97, Ireland

**Keywords:** HTC, Dairy Waste, Hydrochar, Phosphorus, STRUBIAS, Heavy Metals

## Abstract

**Background: **Hydrothermal carbonization (HTC) of dairy processing waste was performed to investigate the effect of temperature and initial pH on the yield and composition of the solid (hydrochar) and liquor produced. All hydrochars met the EU requirements of organo-mineral solid fertilizers defined in the Fertilizing Products Regulation in terms of phosphorus (P) and mineral content.

**Methods: **Laboratory scale HTC was performed using pressurized reactors, and the products (solid and liquid) were collected, stored and analyzed for elemental composition and nutrient content using Inductively coupled plasma optical emission spectroscopy (ICP-OES), ultraviolet-visible spectrophotometry (UV-Vis) and other analytic techniques.

**Results: **Maximum hydrochar yield (60.67%) was observed at T=180℃ and pH=2.25, whereas the maximum P-recovery was 80.38% at T=220℃ and pH=4.6. The heavy metal content of the hydrochars was mostly compliant with EU limitations, except for Ni at T=220℃ and pH=8.32. Meanwhile, further study of Chromium (Cr) species is essential to assess the fertilizer quality of the hydrochars. For the liquid product, the increase in temperature beyond 200℃, coupled with an increase in initial acidity (pH=2.25) drove P into the liquor. Simultaneously, increasing HTC temperature and acidity increased the concentration of NO _3_^-^ and NH _4_^+^ in the liquid products to a maximum of 278 and 148 mg/L, respectively, at T=180℃ and pH=4.6. Furthermore, no direct relation between final pH of liquor and NH _4_^+ ^concentration was observed.

**Conclusions: **HTC improved the nutrient content of dairy waste, allowing for the production of potential solid organo-mineral fertilizers requiring additional treatment to ensure safe fertilizer application.

## Plain language summary

The following paper addresses a potential technique for the treatment of dairy waste under controlled conditions of pressure and temperature. This treatment is known as hydrothermal carbonization. This technique allows for solving the increasing production of dairy waste in the dairy industry. Simultaneously, hydrothermal carbonization leads to the production of hydrochar, which possesses a potential for fertilizer application. The paper demonstrates through an experimental procedure that the hydrochar complies with the EU fertilizer regulations; particularly with Product Function Category (PFC) 1B-(I) for solid organo-mineral fertilizers. The paper also analyzes the chemical composition of the liquid product and recommends a series of additional steps to further develop both the solid and the liquid products. To sum up, the paper proves that hydrothermal carbonization of dairy waste increases the fertilizer potential of the hydrochar, while extra studies and additional treatment remain necessary to ensure a safe and feasible fertilizer produced from waste.

## Introduction

The increase in human population, alongside the continuously changing lifestyle and dietary habits of humans, is leading to the rise of serious concerns over the ability to balance between sustaining human needs and conserving the environment ( Shete & Shinkar, 2013). In particular, the management of agricultural and industrial waste has elevated into a priority for several countries as studies are expecting an increase of 50% expansion in global food production by the year 2050 ( Luqman & Al-Ansari, 2021). This expansion will not come cheap, as the risks for satisfying such growth include resource depletion, environmental imbalance, and waste disposal ( Atallah *et al*., 2020). The latter presents one of the rising challenges for most agricultural and industrial sectors, including the dairy sector.

The dairy sector is experiencing significant expansion, where the production of milk is expected to reach 977 million tonnes by the year 2025, with an increase of 177 million tonnes from the production recorded in 2019 ( Kozłowski *et al*., 2019). The European Union preserves the top spot as the largest milk producer in the world, with an aggregate 160 million tonnes of milk produced in 2018 ( EDA, 2018). The expansion in milk production is followed by an increase in dairy waste resulting from the different processing streams and additives, which has become one of the major sources of industrial waste in Europe ( Carvalho *et al.*, 2013). Dairy waste presents major challenges for environmental stability, the first of which is the effect of traditional disposal methods on natural sources of water and land. In Ireland, the major route for dairy waste disposal is land spreading, whereas a significant quantity (24%) of the waste is removed by licenced contractors ( Carvalho *et al*., 2013). Therefore, the need for treating dairy waste has become a necessity to avoid its potential risks.

In addition to that, the treatment of dairy waste presents an opportunity to achieve a successful economic and environmental cycle. Dairy processing waste (DPW) was recognized as a potential feedstock of STRUBIAS (struvite, biochar, or incineration ashes) products, which are capable of fertilizer applications and soil amendments ( Shi *et al*., 2021a). Ashekuzzaman *et al.* (2019) revealed that lime treated dissolved air floatation (DAF) dairy sludge possessed a significant nutrient content, in addition to heavy metal content that was below EU limitations. The average Nitrogen (N): Phosphorus (P): Potassium (K) content of dairy sludge was 19.5:65.9:3.9 g/kg respectively ( Ashekuzzaman *et al*., 2019). Similarly, the amount of nitrogen in other types of dairy waste was found to be significant, ranging between 14 to 830 mg/L in another study ( Chokshi *et al*., 2016).

Thus, the treatment of dairy waste not only solves the environmental risks of the dairy industry, but also presents an opportunity for producing valuable products for agricultural applications.

Previous experiments for the treatment of dairy waste have been conducted on laboratory and pilot scales. Hydrothermal carbonization (HTC) presents a promising technique for thermochemical treatment of dairy waste to produce valuable products. Wu *et al.* (2018) performed HTC of dairy manure at different values of temperature, residence time and biomass/water ratio. The results showed that HTC improved the elemental composition of dairy manure and its energy potential, and it facilitated the recovery of valuable nutrients such as N, P and K ( Wu *et al.*, 2018). Similarly, Marin-Batista *et al.* (2020) suggested HTC as a method for improving the energy content of biomass derived from dairy waste ( Marin-Batista *et al*., 2020). Finally, Atallah *et al.* (2020) performed HTC on dairy sludge with different experimental conditions, and their results confirm that HTC elevates the carbon properties in hydrochar and increases the structural and chemical stability of the solid ( Atallah *et al*., 2020).

Based on the above, the following paper investigates the HTC of dairy waste and the effects of different operating parameters on the quality and composition of the solid and liquid products, with particular emphasis on the fertilizer potential of the solid hydrochar. To our knowledge, no previous assessment of hydrochar as a potential fertilizer was performed in reference to EU Regulation 1069/2009.

## Methods

### Feedstock

Dairy sludge was collected from a wastewater treatment plant near Limerick, Ireland. The samples were specifically collected from the post-DAF section, where a combination of several filtered dairy waste streams is present. The moisture content of the sludge was measured using the oven dry method (CEN/TS 15414-1:2010) ( ASI, 2006), and it was found to be 79%. No water addition is required as the high moisture content present in the sludge allows for water to act both as a reactant and a catalyst for HTC reactions.

### Hydrothermal carbonization

Hydrothermal carbonization of dairy sludge was performed using PARR 5523 - Catalogue no. 4500 (100 mL in volume) reactor in the University of Limerick, Ireland. Dairy waste samples were introduced into the reactor vessel through a glass liner. The reactor was tightly sealed to allow for autogenous pressure build-up inside, while the temperature and stirring speed of the mixer were controlled through Honeywell© 900 PID controller software. The reaction temperature was varied between 180°C and 220°C to account for the effect of temperature, while the stirring speed of the mixture was set at 180 rpm. The reaction time for all runs was fixed at two hours, with the starting point assumed to be the time at which the set point temperature is reached.

### Sample labelling

To account for the effects of temperature and pH on the outcome, every HTC run was labelled to address the varying conditions. The reaction temperature was changed across three different values (180, 200, 220°C), while the initial pH was varied across three different values (8.32, 4.6, 2.25), adopted and modified from previous configurations ( Ghanim *et al.*, 2017). Therefore, the product labels were designed according to the following order “Product–Temperature–pH,” where “Product” can either be a hydrochar (HC) or a liquor (L). “Temperature” was replaced by the values of the reaction temperature, and “pH” referred to the initial pH of the introduced sample.

Based on the above configuration, the reactions performed were a total of 9 performed in duplicate.

### Product separation

Upon the termination of the reaction, the wet product was subject to separation through Vacuum Filtration using Whatman© Grade 52 filter paper. The outcome was a solid carbonaceous material called “hydrochar” and a dark brown liquid called “liquor.” The wet hydrochar was then dried in a Mason Technology Universal oven UN30 at 105°C for 24 hours, while the liquor was stored in the fridge at 5°C for further analysis.

### Characterization and analysis

***Hydrochar yield.*** The hydrochar and liquor underwent a series of tests for assessing the quality of the HTC reaction and its products. The hydrochar yield was calculated according to the following equation: 


$$HY=\frac{dry\;mass\;of\;hydrochar}{dry\;mass\;of\;feedstock}\times 100\;(1)$$

***Ash content.*** The ash content of the sludge and their derived hydrochars was measured according to CEN/TS 15403 by the aid of a furnace, where dry solid samples were introduced, and the temperature was raised to 550°C and kept constant for 60 minutes. Understanding the change in ash content is an indicator of the fuel quality of the hydro chars and the extent of the reactions occurring.

***P-analysis.*** Total phosphorus (TP) content in the dairy sludge and hydrochars was measured using an Agilent 5100 synchronous vertical dual view inductively coupled plasma optical emission spectrometer (Agilent 5100 ICP-OES). The recovery of P in hydrochar was calculated according to the following equation:


$$\%P\;Recovery=\frac{TP\;in\;hydrochar\;(g)}{TP\;in\;dairy\;sludge\;(g)}\times 100\;(2)$$

In addition to P, the concentration of minerals such as Calcium (Ca), Iron (Fe), Magnesium (Mg) and Sodium (Na) and heavy metals such as Cadmium (Cd), Chromium (Cr), Cobalt (Co), Manganese (Mn), Nickel (Ni) and Zinc (Zn) were also measured using ICP-OES to assess the fertilizer quality of the hydrochar as a potential STRUBIAS product.

***Liquid product.*** P content, as well as ammonia (NH _4_^+^-N) and nitrate (NO _3_^-^) contents in the HTC liquor were measured by UV spectrophotometry using a Hach© DR3900 spectrophotometer. Details of the method are provided in the *Extended data* ( Khalaf, 2022b). The final pH of the HC liquors was measured using a calibrated EU 6+ pH meter, with all measurements performed in duplicate.

## Results and discussion

### Hydrochar yield

**Figure 1.**
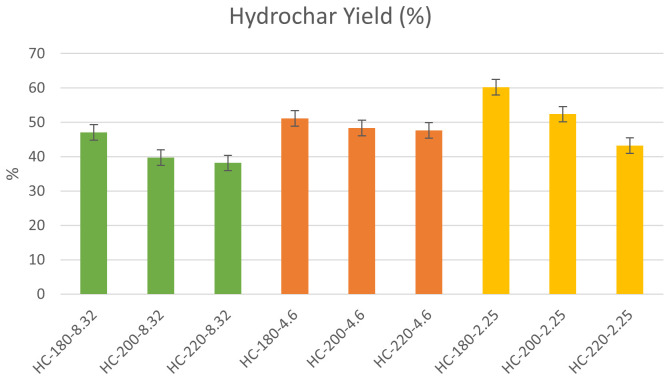
Hydrochar yield at different initial conditions (HC=Hydrochar).

The hydrochar yields reported on a dry basis are presented in Figure 1 for the different initial reaction conditions ( Khalaf, 2022a). Additional data on hydrochar yield calculations are present in Table S1 in the *Extended data* ( Khalaf, 2022b).

***Optimal conditions.*** Since the hydrochar represents the potential product for fertilizer application, it is necessary to identify the optimal operating conditions for maximizing its yield, which was recorded at T=180°C and pH=2.25, suggesting that moderate conditions of temperature and elevated acidity allow for higher solid recovery through HTC.

***Effect of temperature.*** Reaction temperature is considered to be the dominant parameter influencing the extent of reactions occurring through HTC ( Khan *et al.*, 2021). In general, there is an inverse relation between temperature and hydrochar yield, *i.e.,* an increase in temperature leads to a decrease in hydrochar yield. This is because the increase in temperature provides additional energy for further hydrolysis and cleavage of bonds in the structures of the feedstock ( Nizamuddin *et al*., 2017). The effect of temperature on hydrochar yield is consistent for a variety of HTC feedstock. According to Sun *et al.* (2010), the recovery of solid from HTC products was highest at temperatures below 200°C, whereas an increase of temperature to 200-250°C led to a significant increase in liquid yield ( Sun *et al.*, 2010). The decrease in hydrochar yield beyond 200°C is attributed to increased dehydration reactions. This leads to further decomposition of sugars into organic acids and phenolic derivatives comprising the liquid product ( Sharma *et al*., 2020). For this reason, specifying a range of optimal operating temperatures is an important step in optimizing the hydrochar yield.

The experimental results from this study show that the increase in HTC temperature led to a decrease in hydrochar yield for all initial conditions of acidity. At pH=8.32, the increase in HTC temperature from 180°C to 220°C led to a reduction in hydrochar yield from 47.75% to 38.18%. Similarly, at pH=2.25, the effect of temperature on hydrochar yield was more significant, as the yield decreased from 66.27% to 45.53% % upon the increase of temperature from 180°C to 220°C. These results confirm the findings of previous studies. Wu *et al.* (2018) reported similar results from the HTC of dairy manure, where the increase in temperature from 150 to 270°C led to a decrease in hydrochar yield from 74.41% to 52.75% ( Wu *et al*., 2018). Similarly, Xiong *et al.* (2019) performed HTC of swine manure at various temperatures, and the results showed a decrease in hydrochar yield from 58.7% to 50.2% ( Xiong *et al*., 2019). The same trend was reported by Sermyagina *et al.* (2015) during the HTC of wood chips, where an increase in temperature from 180°C to 250°C resulted in a 20% reduction in hydrochar yield ( Sermyagina *et al.*, 2015). 

***Effect of initial pH.*** The effect of initial pH on hydrochar yield is not fully understood. In general, acids are considered to be catalysts which enhance hydrothermal decomposition reactions during HTC ( Flora *et al.*, 2013).

The results of the experiments showed that the effect of initial acidity varied at different reaction temperature, which may reflect the dominant role of reaction temperature during hydrothermal carbonization, as presented in Figure 1. At T=180°C, the increase in initial acidity led to an increase in hydrochar yield with a maximum of 60.27% at pH=2.25. Similarly, at elevated temperatures, the increase in initial acidity led to a significant increase in hydrochar yield with maximum yields obtained at pH=2.25 at T=200°C and 220°C, respectively. In general, it can be observed that an increase in acidic conditions led to a significant increase in hydrochar yield.

The effect of acidic activity on hydrochar yield is not consistent in literature. Our results are compatible with the findings of Ghanim *et al.* (2017) in their HTC experiments on poultry litter. The increase in initial acidity from pH=7 to pH=2 led to an increase in hydrochar yield from 23.91% to 38.13% ( Ghanim *et al*., 2017). Similar findings were reported by Wang *et al.* (2017) during HTC of sewage sludge, where the decrease in initial pH from 11 to 3 led to an increase in hydrochar yield from 61.14% to a maximum of 68.39% ( Wang *et al*., 2017). However, other studies report an opposite effect of initial pH on hydrochar yield. Liu *et al.* (2020) investigated the effect of feed-water pH on the properties of hydrochar produced from sewage sludge, and the results show that an increase of initial pH from 2 to 12 led to a slight and variable increase in hydrochar yield from 52.27% to a maximum of 57.37% at pH=9, after which the yield drops to 53% at pH=12 ( Liu *et al*., 2020). Similarly, Mumme *et al.* (2011) reported an increase in hydrochar yield from the HTC of digested maize silage when the initial pH was increased from 3 to 7 ( Mumme *et al*., 2011).

The reason behind these contradictory findings could lie in the varying extents of decomposition for different HTC experiments. For instance, acidic conditions enhance hydrolysis of initial compounds such as cellulose and hemicellulose into sugars, furans and other phenolic compounds. The re-polymerization and aromatization of these compounds leads to the production of the complex carbon structure known as “hydrochar”. However, if the re-polymerization is inhibited or overcome by side reactions, these products will add to the present liquid complex known as the “HTC liquor”, thus yielding more liquid product. The effect of acidity on the final re-polymerization and aromatization step has not been completely identified.

### Ash content

Ash is composed of the inorganic remains that are left over after the combustion of the material ( Hashan *et al.*, 2013). Understanding the ash content of hydrochar provides insight into the mineral composition of the solid product, in addition to the fuel quality of the product.

The ash content of the initial dairy processing sludge was 48.83%, which is high in comparison to that in other types of feedstock such as orange peel, which was around 13.5% ( Burguete *et al*., 2016). Kwapinska & Leahy (2017) investigated the properties of sludge collected from milk processing plants in Ireland and reported that the typical ash content was 36.41% ( Kwapinska & Leahy, 2017). The high ash content in the dairy processing sludge is due to the addition of ferric coagulants such as FeCl _3_ and FeSO _4_ in the waste water treatment plant reduce the Chemical Oxygen Demand (COD) and Biochemical Oxygen Demand (BOD) levels in dairy wastewater ( Davarnejad *et al.*, 2018). This explanation is supported by the high Fe concentration (114422 mg/kg) present in the dairy waste used for HTC experiments.

**Figure 2.**
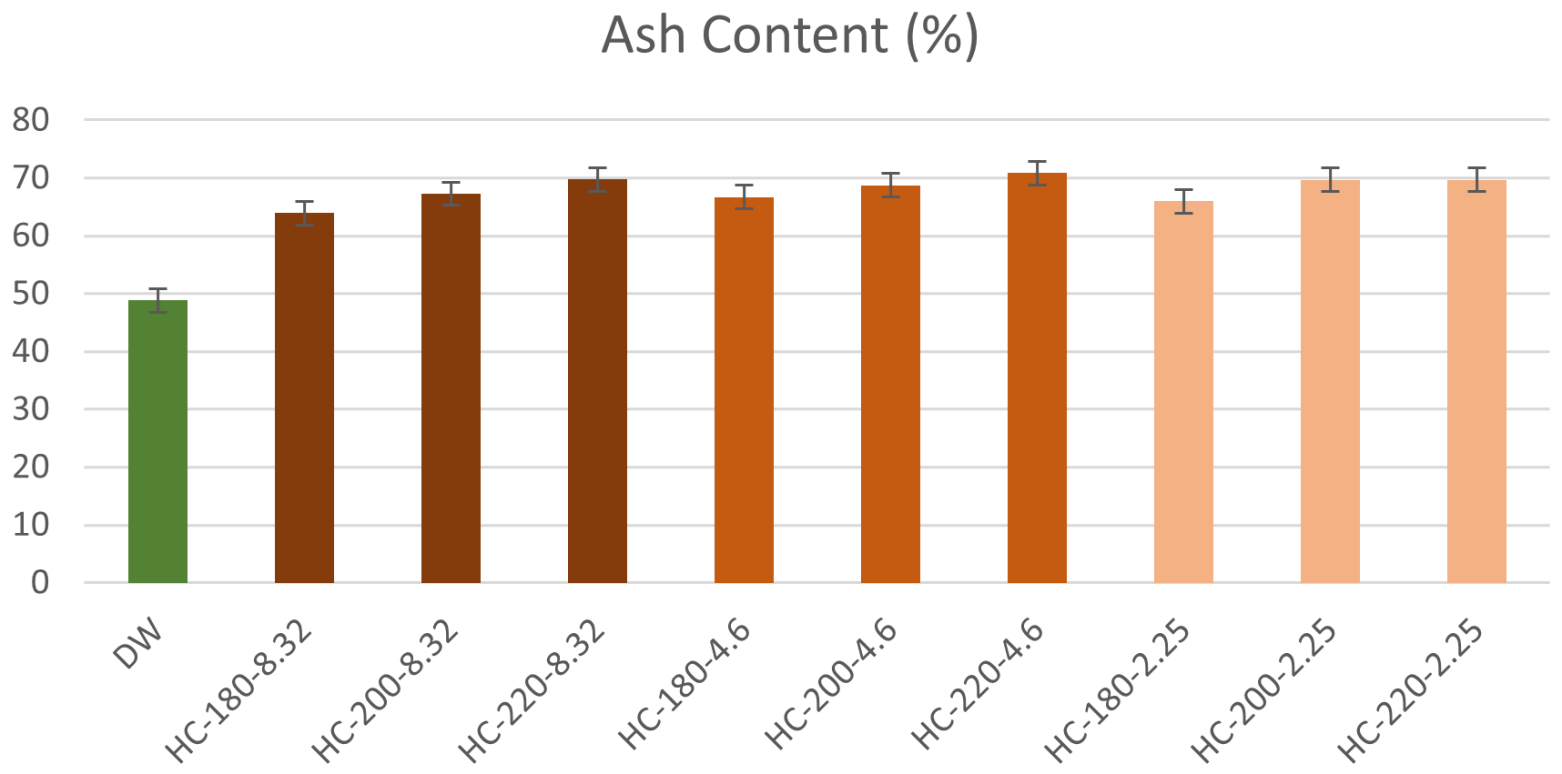
Ash Content of hydrochars from different reaction conditions (DPW=Dairy Processing Waste; HC=Hydrochar).

The ash content of the hydrochars increased following HTC with a minimum increase of 30%, as shown in Figure 2. This result confirms the findings of Garlapalli *et al.* (2016) regarding the increase in ash content of hydrochars formed from digestate at temperatures above 180°C. The authors contributed the increase in ash content to the formation of oxides, which tend to resist melting and remain in the ash ( Garlapalli *et al.*, 2016) The increase in temperature influenced an increase in the ash content in hydrochars with different initial pH. For instance, at pH=8.32, the increase in temperature from 180°C to 220°C led to an increase in ash content from 63.92% to 69.76%. This observation confirms the suggestion of Mäkelä *et al.* (2015) on the significant positive effect of temperature on hydrochar ash content ( Mäkelä *et al.*, 2015). Similar results were reported by Lin *et al.* (2015), where the ash content of hydrochars increased from 54.81% to 63.78% as the HTC temperature increased from 180°C to 270°C ( Lin *et al*., 2015). Meanwhile, the effect of initial pH on ash content could be studied by assessing the fractionation of the initial ash in HTC products, which is presented in Table 1. In the absence of acid addition, the percentage of ash content in hydrochar fluctuated between 53.99% and 68.77%. However, as initial pH decreased, the percentage of initial ash content retained in the hydrochar increased significantly to reach 89.54% at T=180°C and pH=2.25. These results suggested that the hydrochars produced from dairy waste possess low fuel quality due to their high mineral content.

**Table 1.** Ash content and ash fraction in Hydrochar (%).

Sample	Ash Content (%)	Ash Fraction in HC (%)
HC-180-8.32	63.92	53.99
HC-200-8.32	67.27	56.23
HC-220-8.32	69.76	68.77
HC-180-4.6	66.69	84.81
HC-200-4.6	68.72	77.84
HC-220-4.6	70.81	70.58
HC-180-2.25	65.97	89.54
HC-200-2.25	69.69	82.71
HC-220-2.25	69.65	64.95

HC=Hydrochar

### Phosphorus recovery

**Figure 3.**
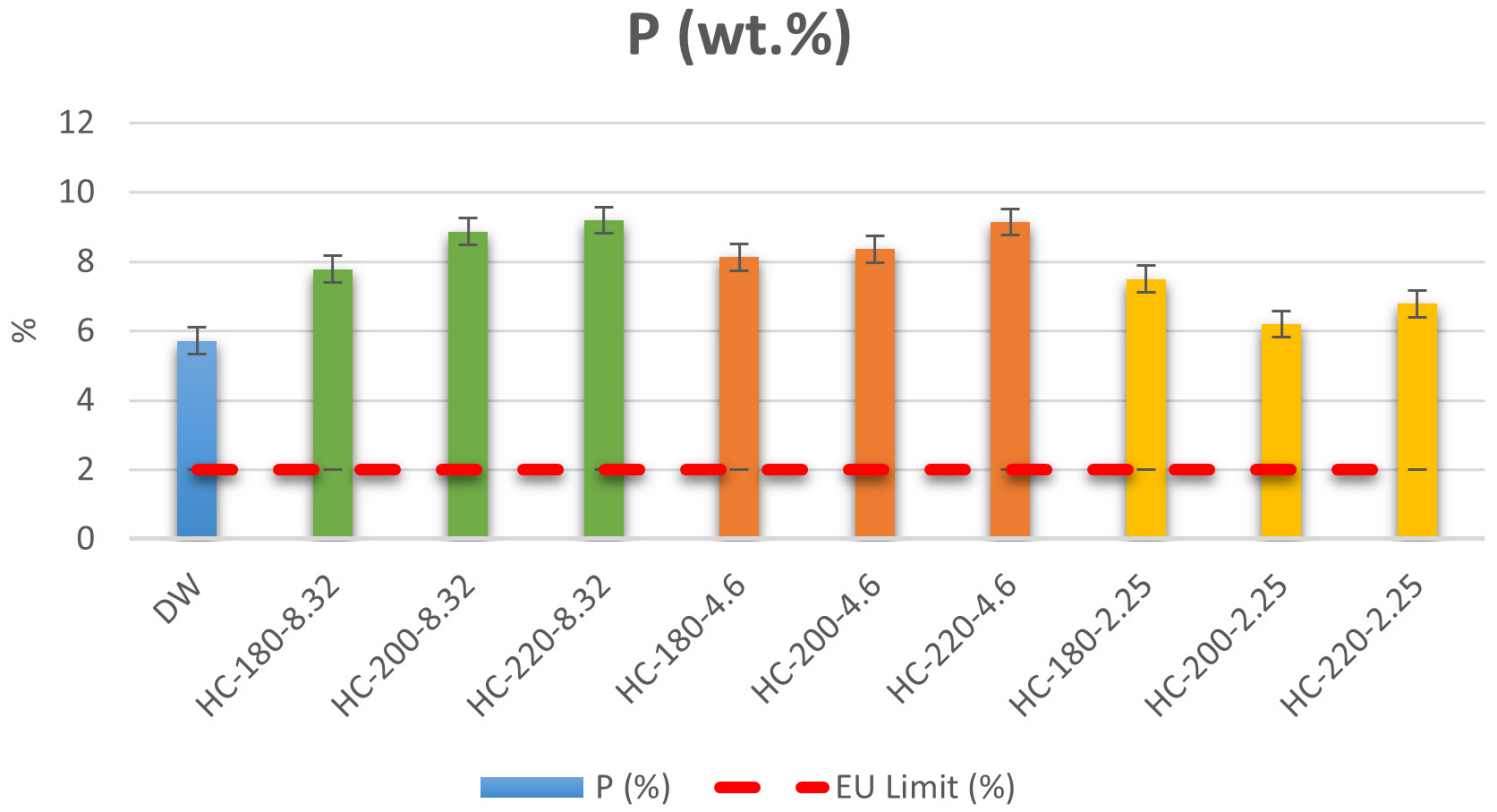
Weight percentage (%) of P in dairy waste and different hydrochars (DW= dairy waste; HC= hydrochar).

***EU regulation.*** One of the approaches for measuring the potential of hydrochar as a fertilizer component is its phosphorus content. According to Product Function Category (PFC) 1 (B).(I) specified in Annex I in EU Regulation 1069/2009, a solid organo-mineral fertilizer consisting of multiple nutrients must contain at least 2% by mass of total P ( OJEU, 2019). Results of the ICP measurement showed that the initial dairy waste contains 5.71% of P on a dry basis. Even though this concentration is above the minimum EU regulation requirement, HTC was used to improve the quality of the dairy waste by producing hydrochar. As shown in Figure 3, the P content in all hydrochars was higher than the initial P content in dairy waste, which demonstrates the ability of HTC to increase the nutrient concentration of dairy sludge. In addition, the increase in temperature led to an increase in the concentration of P for all conditions except at pH=2.25, where the increase in temperature led to a reduction in P content from 7.49% at T=180°C to 6.78% at T=220°C. These results confirmed the observations of Shi *et al.* (2019) regarding the positive effect of temperature on the concentration of P in hydrochar. The study also suggested that high concentrations of acid addition can drive phosphorus into the liquid product, which explains the reduction of P percentage at initial pH of 2.25 ( Shi *et al.*, 2019). This suggestion was confirmed in this paper, where an increase in temperature led to a reduction in P-concentration in the liquid product.

In terms of EU regulations, the P percentage in all hydrochars was higher than the regulation limit, with a maximum P percentage=9.14% at T=220°C and pH=4.6. Thus, the produced hydrochars qualify for solid organo-mineral fertilizer application in terms of P content.

**Figure 4.**
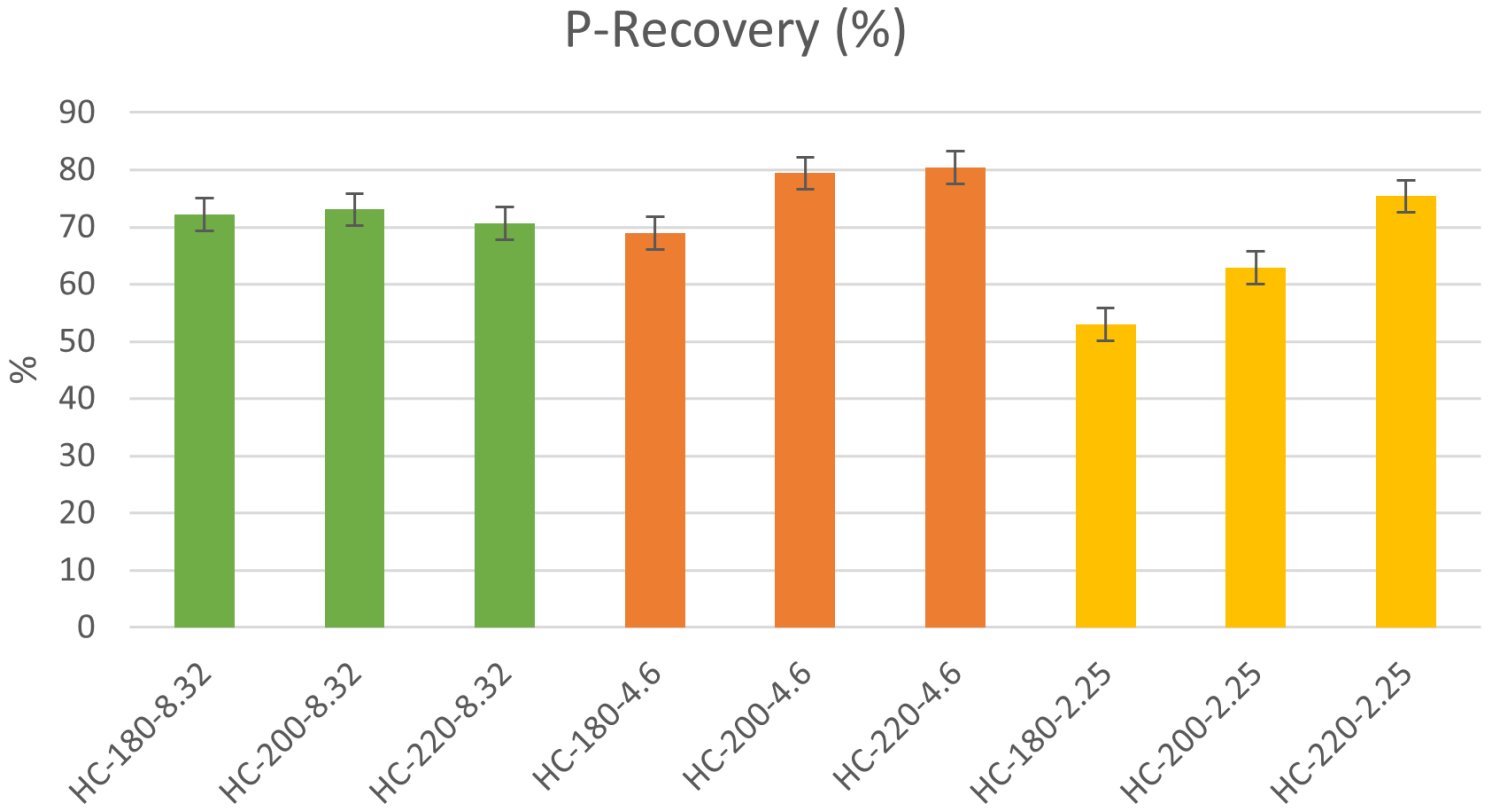
P-recovery from hydrochars at different conditions of temperature and pH.

***P-recovery.*** The recovery of phosphorus in hydrochar has been emphasized by several previous studies focusing on the immobilization of P-species through HTC ( Dai *et al*., 2015). In general, the definition of P-recovery may vary, yet in this paper, it refers to the amount of P that was retained in hydrochar. Data for P-recovery calculations are present in Table S2 in the *Extended data* ( Khalaf, 2022b). Previous studies showed that the recovery of P in hydrochars was found to be around or above 80% ( Meng *et al.*, 2019). As shown in Figure 4, this study found that the P-recovery in hydrochar produced from HTC of dairy waste was between 53% and 80% with maximum P-recovery of 80.23% observed at T=220°C and pH=4.6. This further showed that HTC favored the concentration of P in the hydrochar, which concurs with the previous findings of Heilmann *et al.* (2014), where up to 90% of the total P was recovered in the hydrochar resulting from the HTC of different animal manures ( Heilmann *et al*., 2014). Similarly, Huang & Tang (2016) investigated the recovery of P from activated sludge and anaerobically digested sludge, and the results showed that 89.3% and 95.5% of P was recovered in the hydrochar produced from each feedstock, respectively ( Huang & Tang, 2016).

**Figure 5.**
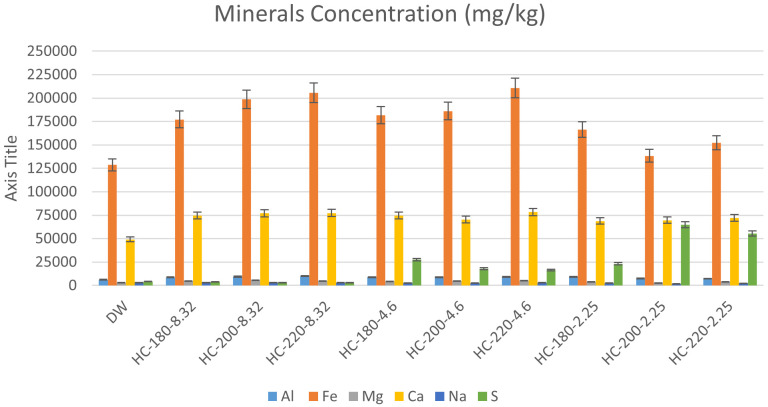
Minerals concentration (mg/kg) in hydrochars produced at different conditions.

***Effect of temperature and initial pH.*** Similar to its effect on hydrochar yield, temperature plays a significant role in influencing the recovery of P in hydrochar. In the absence of acid addition, the P-recovery in hydrochar was almost consistent and fluctuated between 70% and 72%. However, the effect of temperature became more significant with pH reduction through acid addition. At a pH=4.6, the increase in temperature from 180°C to 220°C led to an increase in P-recovery from 69% to 80%. Similarly, and more significantly, at pH=2.25, the same increase in temperature produced a 42.58% increase in P-recovery. These results showed that the effect of temperature alone on P-recovery was not significant; however, in the presence of acid addition, the recovery of P from hydrochar increased significantly as temperature was increased. To explain the effect of temperature, Ekpo *et al.* (2016) suggested that the precipitation of P with metals such as Ca, Mg and Fe leads to the accumulation of P in the hydrochar ( Ekpo *et al.*, 2016). The increase in temperature increases the concentration of metal ions available for P-precipitation, which explains the increase in P-recovery at higher temperatures. Zheng *et al.* (2022) showed that an increase in the HTC reaction temperature of sewage sludge from 160°C to 280°C resulted in a significant increase in P-concentration in the hydrochar from 38.92 mg/g to 46.99 mg/g, ( Zheng *et al*., 2022). The results of mineral concentrations from ICP-OES, in this study, support this suggestion, since all hydrochars possessed increased concentrations of minerals compared to the original dairy waste. As shown in Figure 5, increasing HTC temperature led to an increase in the concentrations of Ca, Fe and Mg, with Fe remaining the dominant mineral. The effect of temperature on P-recovery in hydrochar has been reported in literature. Shi *et al.* (2019) showed that an increase in temperature from 170°C to 320°C led to an increase in hydrochar P-content from 81.4% to 95.96% ( Shi *et al*., 2019). Similarly, Cui *et al.* (2020) observed a similar pattern during the HTC of wetland biomass wastes where an increase in HTC temperature improved total P recovery in hydrochar from 68.45% at 200°C to 84.7% at 260°C ( Cui *et al*., 2020).

To assess the effect of initial pH, the results were analyzed by fixing the temperature and varying the initial pH. At moderate temperatures (T=180°C), P-recovery in the hydrochar showed a significant reduction as initial pH decreased from 8.32 to 2.25. However, at higher temperatures, the effect of initial pH on P-recovery was reversed, where an increase in initial acidity enhanced P-recovery, with a maximum observed at pH=4.6. The data suggests that at moderate acidity the effect of temperature is dominant allowing for more precipitation of P in the hydrochar. The effect of initial pH on P-recovery in the hydrochar was investigated by Zheng *et al.* (2020). As the initial pH increased from 3 to 7, the total P enrichment increased from 81.42% to 89.78%, after which it decreased to 85.2% at pH=13 ( Zheng *et al*., 2020). Similar results were reported by McIntosh *et al.* (2022), where the increase in the initial pH of sewage sludge HTC from 2 to 7 led to an increase in P-recovery from 92.8% to 98.4%, respectively. However, an increase in initial pH from 7 to 12 at fixed conditions of temperature led to a decrease in P-recovery from 98.4% to 88.7% ( McIntosh *et al*., 2022).

### Heavy metals

***EU limits.*** Another significant indicator for assessing the fertilizer potential of hydrochar is the concentration of heavy metals, which can limit the application of hydrochars. Heavy metals are defined as the metals and metalloids possessing densities higher than 5g/cm ^3^, and the most common heavy metals in the fertilizer sector include Arsenic (As), Cadmium (Cd), Chromium (Cr), Copper (Cu), Nickel (Ni), Lead (Pb) & Zinc (Zn) ( Cui *et al*., 2021).

The concentrations of heavy metals in the collected dairy waste and hydrochars are shown in Table 1 along with the concentration limits defined by PFC 1 (B). (I) in EU Regulation 1069 for solid organo-mineral fertilizers ( OJEU, 2019).

The results show that the heavy metal concentrations of the initial dairy processing waste were below the limit of contamination specified by the EU Regulation for all heavy metals. Similarly, all of the hydrochars showed concentrations that were below the maximum allowed limits for all heavy metals except for Ni at T=220°C and pH=8.32, which recorded a concentration of 60 mg/kg, slightly above the allowed limit of 50 mg/kg. The total concentration of Chromium (Cr) was also studied, and it was observed that HTC concentrates Cr in hydrochars, which can present a source of environmental pollution ( Ghanim *et al*., 2022). However, assessing the actual implication of Cr concentration in hydrochars compared to EU regulations can be done by Cr speciation. The most abundant heavy metal was Zn (136–230 mg/kg), while As and Cd were below limits of detection for all hydrochars, thus abiding by the requirements for solid organo-mineral fertilizers.

In all hydrochars, the concentration of heavy metals was generally higher than the original feedstock, which shows that the major destination for heavy metals was hydrochar. The percentage of heavy metals that were retained in hydrochars were not lower than 60% for all heavy metals except Pb, which had an average recovery below 50% in the hydrochar. Data on the heavy metal fractions retrieved in the hydrochars are provided in Table S4 in the *Extended data* ( Khalaf, 2022b). Similar observations were reported by Fu *et al.* (2022) during the HTC of livestock manure, where HTC was responsible for enriching hydrochar with heavy metals. The authors explained this phenomenon from two perspectives, the first of which is the low mobility of heavy metals, while the second explanation stems from the porous structure of hydrochar, which enhances the adsorption of heavy metals ( Fu *et al*., 2022).

It was observed that even though HTC concentrates heavy metals in the hydrochar, the latter nevertheless qualified for use as a solid organo-mineral fertilizer. However, further validation of the fertilizer quality of the hydrochars can be performed by assessing the concentration of hexavalent chromium. The optimal hydrochar with the best qualifications for a solid organo-mineral fertilizer in terms of heavy metal concentration was detected at T=200°C and pH=4.6.

**Figure 6.**
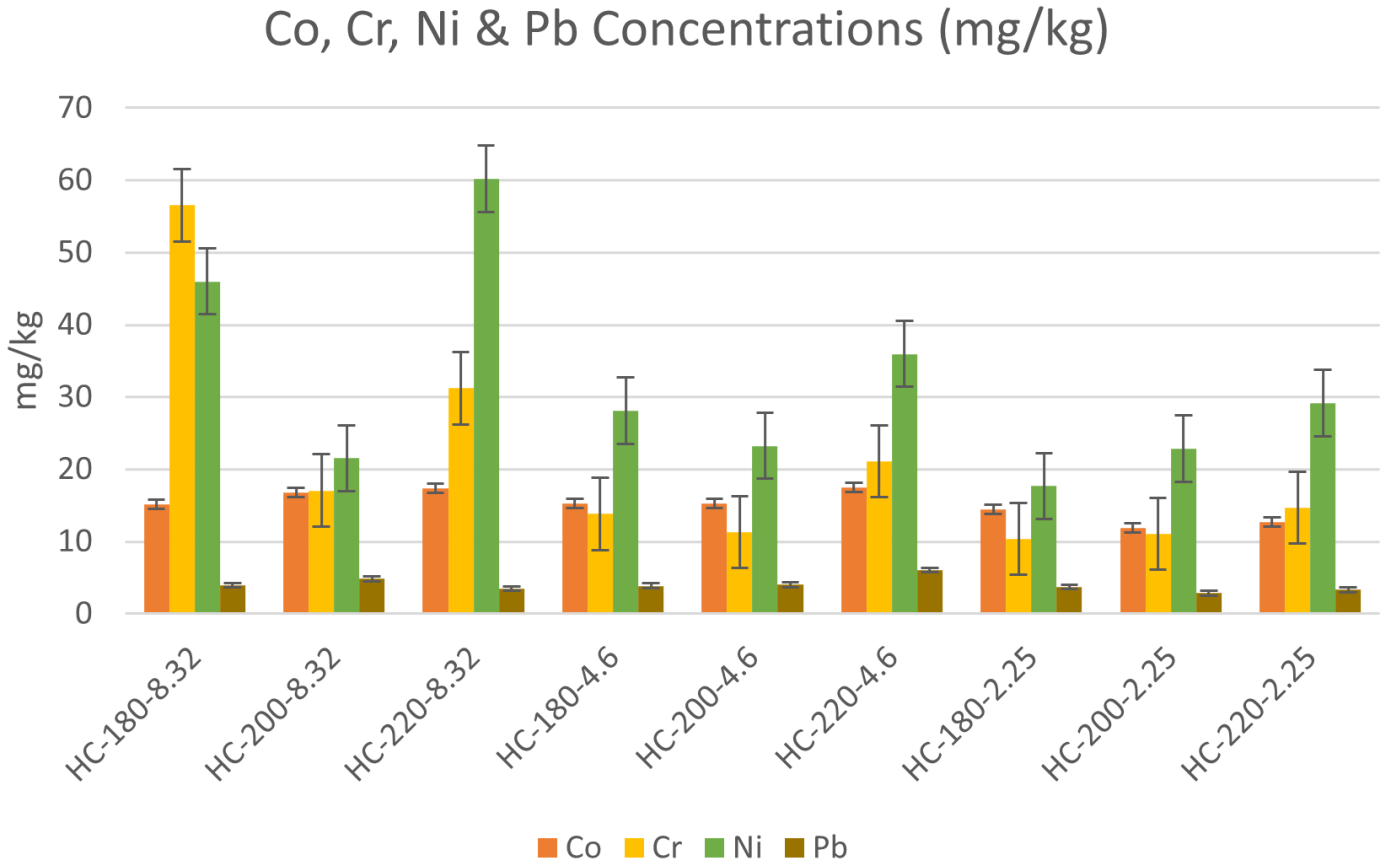
Change in Cobalt, Chromium, Nickel & Lead concentrations for different hydrochars.

**Figure 7.**
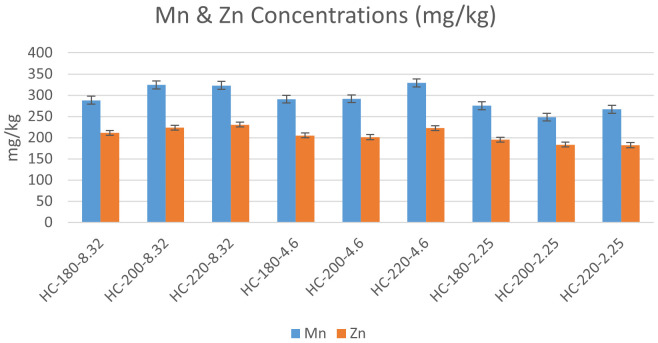
Change in Manganese & Zinc concentrations for different hydrochars.

***Effect of Parameters.*** The effect of temperature and initial pH on heavy metal concentrations was studied according to the results presented in Figure 6 & Figure 7.

In the absence of acid addition (pH=8.32), the concentrations of all heavy metals except Cr experienced an overall increase with an increase in HTC temperature. Similar results were found by Breulmann *et al.* (2017) during the HTC of sludge. As temperature increased from 180°C to 200°C, the concentration of Pb increased from 34 to 40 mg/kg, along with an increase in the concentrations of Zn, Mn, Cr, Cd and As ( Breulmann *et al.*, 2017). This suggests that the effect of temperature on HTC reactions increases the ability for adsorbing heavy metals by increasing the porosity of hydrochars ( Jaruwat *et al.*, 2018).

Upon acid addition, the effect of temperature differed depending on the metal species. At pH=4.6, the increase in temperature resulted in an increase in the concentration of all heavy metals, which confirmed the previous findings of Zhai *et al.* (2016) regarding the effect of acidic media on the accumulation of heavy metals in hydrochar ( Zhai *et al*., 2016). However, as the initial pH was reduced to 2.25, the effect of temperature was reversed for Mn, Zn, Pb, and Co, whereas Ni and Cr maintained the same trend of concentration increase with temperature. One of the suggested explanations for the effect of acidity on heavy metals can be the enhancement of competitive ion exchange due to the increase in H ^+^ ions with acid addition ( Inglezakis *et al.*, 2003). Breulmann *et al.* (2017) studied the effect of initial pH, and their results revealed that acid addition showed different effects on different heavy metals. For Zn, Cr and Cu, the increase in initial acidity reduced the concentration of heavy metals in hydrochar, while an opposite effect was observed on Ni, Pb and Mn upon the decrease in initial pH ( Breulmann *et al*., 2017).

### Liquid product analysis

***Final pH.*** Measuring the final pH of HTC liquor provides an insight into the severity of the reactions occurring during HTC by hinting to the composition of the liquid product ( Borrero-López *et al.*, 2020). Lower values of final pH are usually connected to the production of organic acids such as acetic, citric, lactic and formic acids from the decomposition of sugars and furfurals ( Lu *et al.*, 2013).

As shown in Table 2, the conditions of liquors produced at different reaction conditions were fluctuating between slightly acidic and neutral. The minimum final pH (5.38) was recorded at T=180°C and initial pH of 2.25, whereas the maximum final pH (7.88) was observed at T=200°C and an initial pH of 4.6. In general, the final pH of the different liquors did not show any significant deviation from the normal range of pH presented in literature. Reza *et al.* (2015) revealed that the pH of the liquid product resulting from the HTC of wheat straw at different initial conditions of acidity ranged between 3–10 ( Reza *et al.*, 2015).

**Table 2.** Concentrations of heavy metals (mg/kg) in comparison to EU limits.

Concentration (mg/kg)								
	As	Cd	Cr	Cu	Ni	Mn	Pb	Zn
EU Limit	40	1.5	-	600	50	-	120	1500
DW	0.53	0.1	5.32	4.16	6.96	181.69	4.26	136.03
HC-180-8.32	<1.5	<0.15	56.52	9.55	45.98	288.23	3.93	211.18
HC-200-8.32	<1.5	<0.15	17.06	7.37	21.52	324.17	4.81	223.5
HC-220-8.32	<1.5	<0.15	31.2	8.27	60.21	322.93	3.49	230.49
HC-180-4.6	<1.5	<0.15	13.8	7.14	28.09	290.78	3.86	205.13
HC-200-4.6	<1.5	<0.15	11.3	7.08	23.23	291.53	4	201
HC-220-4.6	<1.5	<0.15	21.13	9.45	35.96	329.16	6.03	222.63
HC-180-2.25	<1.5	<0.15	10.33	7.54	17.68	275.32	3.68	195.25
HC-200-2.25	<1.5	<0.15	11.03	6.49	22.83	248.23	2.83	183.49
HC-220-2.25	<1.5	<0.15	14.69	6.35	29.14	266.83	3.3	182.37

(DW=dairy Waste)

In the absence of acid addition, the pH of the final liquor was lower than that of the initial feedstock for all experiments. Furthermore, the increase in reaction temperature from 180 to 220°C led to an increase in the final pH of the resulting liquor from 5.72 to 7.12, respectively. This reduction in acidity seems to contradict the increasing production of organic acids at elevated temperatures, yet two explanations for the increase in final pH are suggested. The first explanation links the increase in final pH to the production of ammonium products which have a basic chemistry. Ammonia products are the final products of protein hydrolysis, especially that the average concentration of protein in dairy wastewater is estimated to be around 388 mg/L ( Kurup *et al.*, 2019). According to Wang *et al.* (2019), successive degradation of amino acids produces ammonia alkaline groups at high temperature, and these groups are concentrated in the liquid product, thus increasing the basicity of HTC liquor ( Wang *et al.*, 2019).

To address this possibility, the concentrations of NH _4_^+^-N in the liquors were measured through spectrophotometry. In the absence of acid addition (pH=8.32), the concentration of ammonia increased with an increase in reaction temperature, yet this increase was not directly parallel to the increase in final pH. At moderate acidic conditions (pH=4.6), the final pH of the liquors was almost neutral at different reaction temperatures, and the concentration of ammonia was significantly higher than that in the absence of acid addition. Yet, the pattern relating the concentration of ammonia with the final pH was not consistent. Finally, at severe acidic conditions (pH=2.25), the final pH of the liquors became slightly acidic, which is normal due to the presence of higher concentrations in the reaction. Meanwhile, the concentrations of ammonia were similar to those recorded at moderate initial acidity, with similar inconsistency between the concentrations and the final pH of the liquor.

The results show that ammonia products indeed played a major role in increasing the final pH of HTC liquor, especially under acidic initial conditions, which enhance the release of ammonia from biomass ( Inoue *et al.*, 1997). However, the lack of direct correlation between the increase in final pH and the ammonia concentration in the liquor suggests that several different factors influence the increase in final pH. Similar observations were reported by Dai *et al.* (2017) during the HTC of cattle manure at different conditions of initial acidity. Their results showed that no direct correlation was found between the concentration of NH _4_^+^-N concentration and the final pH of aqueous products ( Dai *et al*., 2017).

Another explanation for the increasing pH of the liquid product comes from the facilitation of dehydration reactions at elevated temperatures, which increases the water in the liquid product, thus diluting its acidic composition ( Ghaziaskar *et al.*, 2019).

***Total phosphorous concentration.*** The concentration of total phosphorous (TP) in the Hydrochar (HC) liquors produced at varying initial conditions was measured. The results showed that at different conditions of initial pH, the concentration of TP decreased with an increase in reaction temperature, which can be explained by the simultaneous increase in P-recovery in the hydrochar as shown earlier in Figure 4. Since higher temperatures favor P-precipitation in the solid product, the concentration of P in the liquid product was expected to decrease. This was observed by Cui *et al.* (2021) during the HTC of wetland biomass, where an increase in HTC temperature from 200 to 260°C led to a significant and gradual decrease in P concentration in the liquid product from 492.73 mg/L to 36.7 mg/L, respectively ( Cui *et al.*, 2019). These findings support the results of our study, where the decrease in initial pH from 8.32 to 2.25 led to a significant increase in TP concentration in liquor at all reaction temperatures, as shown in Table 3. The extraction of P into HTC liquor at different conditions of initial pH was investigated by Ekanthalu *et al.* (2021), and their results confirmed the significant increase of P recovery in the liquor following the addition of inorganic acid ( Ekanthalu *et al.*, 2021). Similar observations were reported by Shi *et al.* (2021b) regarding the influence of acidic media on the release of P to HTC liquor ( Shi *et al*., 2021b). This increase can be explained by the increase in the production of the liquid, which drives a portion of the phosphorus into the liquor. The increase in average liquor yield from 76% to 82% as pH decreases from 8.32 to 2.25 supported this suggestion.

**Table 3.** Liquid Yield, final pH and Ammonia concentrations of Hydrothermal Carbonization liquors.

Sample	Temperature (°C)	Initial pH	Liquor Yield (%)	Final pH	NH4 _4_^+^-N (mg/L)	NO _3_^-^ (mg/L)	PO _4_^3-^ (mg/L)
**L1**	180	8.32	69.022	5.72	81.67	170.77	133.16
**L2**	200	8.32	78.17	6.48	56.07	299.69	119.25
**L3**	220	8.32	80.85	7.12	135.11	185.63	82.74
**L4**	180	4.6	75.69	7.01	147.92	278.01	132.15
**L5**	200	4.6	79.25	7.88	94.68	255.71	118.24
**L6**	220	4.6	78.21	7.665	106.24	202.28	111.15
**L7**	180	2.25	81.94	5.385	99.85	161.46	168.51
**L8**	200	2.25	82.77	6.13	192.71	172.46	143.62
**L9**	220	2.25	79.43	6.71	38.57	197.94	168.28

**Figure 8.**
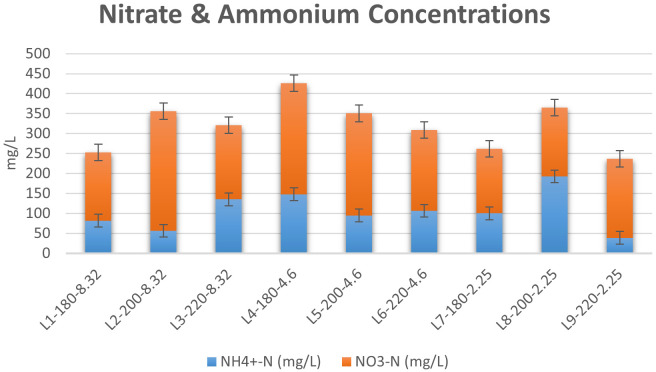
Concentrations of nitrate and ammonium in Hydrothermal Carbonization liquors from different conditions.

***Nitrate and ammonium concentration.*** In addition to estimating the fertilizer quality, assessing nitrogen content in HTC products provides insight into the extent of decomposition reactions occurring. According to literature, the dominant N-species in bio-oil are NH _4_^+^-N and NO3-N, along with minor presence of NO _2_^—^N and CN ^—^N ( Kruse *et al.*, 2016). The results of the spectrophotometric analysis are presented in Figure 8. It was clear that the concentration of nitrate was higher than that of ammonium for almost all liquors produced, which is in agreement with the findings of previous investigations ( Shrestha *et al.*, 2021). The effects of initial conditions on inorganic N content were also investigated. The effect of temperature on inorganic nitrogen content varied at different conditions of initial acidity. In the absence of acid addition, an increase in temperature from 180°C to 220°C increased the N-content in liquor, with a peak concentration of 355.76 mg/L at T=200°C. Furthermore, the increase in temperature expanded the gap between NO _3_^-^ and NH _4_^+ ^concentrations, where an increase in temperature from 180°C to 200°C led to an increase in NO _3_^–^ concentration from 170 to 300 mg/L, accompanied with a decrease in NH _4_^+^ concentration from 82 to 56 mg/L. This effect was reversed as the temperature was increased to 220°C. Following acid addition, the effect of temperature became more significant. At pH=4.6, the increase in temperature induced a large decrease in ammonia concentration, whereas the concentration of nitrate decreased slightly. However, at pH=2.25, ammonia concentration increased from 100 to 193 mg/L when the temperature was increased from T=180°C to T=200°C, followed by a large decrease to 39 mg/L at T=220°C.

The results reflected previous suggestions on the possible pathways of nitrogen during HTC. The increase in inorganic N content upon mild increase in temperature can be attributed to two major routes, the first of which is the breaking of solid nitrogen structures upon temperature increase, which drives the nitrogen into aqueous compounds. However, the elevation of HTC temperature to 240°C favors cracking of nitrogen into light gas, which will eventually decrease N content in the liquid product ( Xiao *et al*., 2019). The second route explaining the increase in inorganic nitrogen content in HTC liquor was proposed by Xiao *et al.* (2019), which is the hydrolysis of Protein-N into inorganic N upon mild increase in HTC temperature ( Xiao *et al.,* 2019). Furthermore, the results confirm previous findings regarding the effect of temperature on the speciation of inorganic N in HTC liquors. Fu *et al.* (2022) reported a significant decrease in NH _4_^+^ upon an increase in temperature, yet the decrease in NO _3_^-^ concentrations was not significant ( Fu *et al*., 2022). Similar results were reported by Alhnidi *et al.* (2020) during the HTC of cattle manure ( Alhnidi *et al.*, 2020). Therefore, it can be deduced that HTC enhanced the concentration of inorganic N in the liquid product, which eventually avoids the polluting effect of nitrate concentration ( Peña-Haro *et al.*, 2010) in hydrochar as a potential fertilizer.

It is worth noting that the highest concentration of nitrates and ammonia combined was found to be 425.93 mg/L at T=180°C and pH=4.6, which was similar to the optimum conditions observed in literature ( Shrestha *et al*., 2021).

## Conclusions

In this paper, hydrothermal carbonization of sludge from a dairy processing factory was performed in order to produce materials which could be valorized as fertilizer components. The effects of temperature and initial pH on the composition and quality of the products were investigated. It was found that increasing temperature enhances the production of hydrochar with increased fertilizer potential in terms of P-content, mineral and heavy metal content with the exception of Cr, which requires further studying. The optimal conditions for the fertilizer component quality hydrochar were at moderate temperatures (180–200°C) and moderate acidity (pH=4.6). Also, temperature and increased acidity enhanced the concentration of inorganic nitrogen and phosphorus in HTC liquor. Finally, no direct correlation between final pH of liquors and corresponding NH _4_^+^ concentration was observed.

## Data availability

### Underlying data

Zenodo: Underlying Data for Manuscript titled "Hydrothermal Carbonization (HTC) of Dairy Waste: Effect of Temperature and Initial Acidity on the composition and quality of solid and liquid products. https://doi.org/10.5281/zenodo.6647010 ( Khalaf, 2022a).

This project contains the following underlying data:

- REFLOW Heavy Metals Calculation-Underlying Data.csv
- REFLOW Phoshporus Recovery Calculations-Underlying Data.csv

### Extended data

Zenodo: Extended Data for Manuscript titled "Hydrothermal Carbonization (HTC) of Dairy Waste: Effect of Temperature and Initial Acidity on the composition and quality of solid and liquid products" https://doi.org/10.5281/zenodo.6584574 ( Khalaf, 2022b).

This project contains the following extended data:

- Document summarizing all extended calculations and method descriptions, such as:
  - Table S1: Hydrochar Yield and final pH calculations
  - Table S2: Phosphorus Recovery Calculations
  - Table S3: Experimental Data for pH adjustment
  - Table S4: Weight Percentage (%) of heavy metals in hydrochars
  - Table S5: Liquor Yield Calculations (mass basis)
  - DR3900 UV-Vis Spectrophotometer Methods
    ▪ LCK350
    ▪ LCK303
    ▪ LCK340

Data are available under the terms of the Creative Commons Attribution 4.0 International license (CC-BY 4.0).

## Ethics and consent

Ethical approval and consent were not required.
