# Supplementary material for: Hydrothermal carbonization (HTC) of dairy waste: effect of temperature and initial acidity on the composition and quality of solid and liquid products
Source: Open Res Eur. 2023 Sep 6;2:83. Originally published 2022 Jun 23. [Version 3] doi: 10.12688/openreseurope.14863.3 (PMC10445854; doi:10.12688/openreseurope.14863.3)
Supplement: Peer review history

# Reviewer report: Reviewer response for version 3

**DOI:** 10.21956/openreseurope.17655.r34943

**Published:** 2023-10-31

**Authors:** Rafal Marcin Lukasik

**Affiliations:** 1 Łukasiewicz Center, Warsaw, Mazowieckie, Poland

The authors implemented suggested changes and this work is ready to be indexed in the present form.

Is the study design appropriate and does the work have academic merit?

Partly

Is the work clearly and accurately presented and does it cite the current literature?

Yes

If applicable, is the statistical analysis and its interpretation appropriate?

No

Are all the source data underlying the results available to ensure full reproducibility?

Yes

Are the conclusions drawn adequately supported by the results?

Yes

Are sufficient details of methods and analysis provided to allow replication by others?

Yes

Reviewer Expertise:

Chemical Engineering

I confirm that I have read this submission and believe that I have an appropriate level of expertise to confirm that it is of an acceptable scientific standard.

# Reviewer report: Reviewer response for version 3

**DOI:** 10.21956/openreseurope.17655.r34944

**Published:** 2023-10-31

**Authors:** Elena Díaz

**Affiliations:** 1 Universidad Autonoma de Madrid, Madrid, Community of Madrid, Spain

The quality of the revised manuscript has significantly improved.  However, I include some some specific aspects/questions/mistakes to be considered by the authors.

- Page 11: “ *The effect of temperature and initial pH on heavy metal concentrations was studied according to the results presented in* *Table 2* *”*; I think that Table 2 has to be changed by Table 4.
- Page 12: “ *As shown in* *Table 3* *, the conditions of liquors produced at different reaction conditions were fluctuating between slightly acidic and neutral*.” I think that Table 3 has to be changed by Table 5.
- Table 5 and Figure 8 include the same information. I consider that Figure 8 can be removed.

- Page 11: The authors should explain why a lower specific area improves the adsorption of heavy metals.
- The data in the tables should be accompanied by their corresponding error.

Is the study design appropriate and does the work have academic merit?

Partly

Is the work clearly and accurately presented and does it cite the current literature?

Partly

If applicable, is the statistical analysis and its interpretation appropriate?

Partly

Are all the source data underlying the results available to ensure full reproducibility?

Yes

Are the conclusions drawn adequately supported by the results?

Yes

Are sufficient details of methods and analysis provided to allow replication by others?

Partly

Reviewer Expertise:

1. Environmental technologies focused on the wastewatertreatment by means of advanced catalytic and biological processes and wasterecovery; 2. Preparation and application of carbonmaterials from biomass wastes for water treatment and in waste-to-energyconversion and biomass pretreatment for bioenergy; 3. Hydrothermal carbonization of biomass wastes coupling to anaerobic digestion to carry out the valorization of these wastes from a material (synthesis of biocarbon, soil improvers, nutrient recovery, synthesis- of fertilizers) and energetic (solid biofuel, biogas, biohydrogen) point of view.

I confirm that I have read this submission and believe that I have an appropriate level of expertise to confirm that it is of an acceptable scientific standard.

# Reviewer report: Reviewer response for version 2

**DOI:** 10.21956/openreseurope.16166.r31931

**Published:** 2023-06-28

**Authors:** Dimitris Kalderis

**Affiliations:** 1 Hellenic Mediterranean University, Chania, Greece

The submitted manuscript describes the HTC of dairy waste and the analysis of the products, focusing on P and N recovery. The text is well-structured and the characterization strategy is appropriate for the objectives of the work. The results are clearly written and compared with existing literature. HTC of dairy waste is less studied compared to the HTC of lignocellulosic biomasses, therefore it has increased significance, especially for European countries with high dairy production. I recommend indexing of the manuscript after the following comments are taken into consideration: 

 1. The authors should add (preferably in the Introduction) that the presence of phytotoxic organic substances (such as furfurals and organic acids) in the hydrochars prevent the widespread use of the product in agriculture. Therefore, it is not only a matter of nutrients, but methodologies should be developed to eliminate toxic phytotoxic substances from hydrochars. The authors can use the following publications to support this

 https://doi.org/10.1016/j.chemosphere.2022.134357 ^1^

 https://doi.org/10.1007/s11356-021-12517-5 ^2^

 2. How did the authors adjust the pH value of the feedstock before HTC? Please add this info in the methods section.

 3. If a full analysis of the dairy sludge composition is available it should be added in the Methods-feedstock section.

Is the study design appropriate and does the work have academic merit?

Yes

Is the work clearly and accurately presented and does it cite the current literature?

Yes

If applicable, is the statistical analysis and its interpretation appropriate?

Yes

Are all the source data underlying the results available to ensure full reproducibility?

Yes

Are the conclusions drawn adequately supported by the results?

Yes

Are sufficient details of methods and analysis provided to allow replication by others?

Yes

Reviewer Expertise:

Hydrochar, hydrothermal carbonization, biomass, biochar

I confirm that I have read this submission and believe that I have an appropriate level of expertise to confirm that it is of an acceptable scientific standard.

## References

[1] : Recent progress on the phytotoxic effects of hydrochars and toxicity reduction approaches. *Chemosphere* .2022;298: 10.1016/j.chemosphere.2022.134357 134357 10.1016/j.chemosphere.2022.134357 35313162

[2] : The effect of organic solvent washing on the structure of hydrochar-based dissolved organic matters and its potential environmental toxicity. *Environ Sci Pollut Res Int* .2021;28(21) : 10.1007/s11356-021-12517-5 26584-26594 10.1007/s11356-021-12517-5 33484455

# Reviewer report: Reviewer response for version 2

**DOI:** 10.21956/openreseurope.16166.r32468

**Published:** 2023-06-21

**Authors:** Elena Díaz

**Affiliations:** 1 Universidad Autonoma de Madrid, Madrid, Community of Madrid, Spain

The manuscript describes the hydrothermal carbonization of dairy sludges evaluating the effect of temperature (180 – 220 ºC) and initial pH (2.25 – 8.32) on hydrochar yield, fate of P, N and metals in order to obtain a producer with value as fertilizer. The following comments address some specific aspects/questions to be considered by the authors.

 Abstract:

- The aim of the work is to obtain a solid fertilizer, then the composition of the liquid product is not essential in the abstract.
- Why do the authors say that “HTC improved the nutrient content of dairy waste”? Is the nutrient content concentrated in the solid product? More specific information should appear in the abstract.

Methods:

- It will be convenient to include all the characteristics of the feedstock in this section (moisture, C content, pH, main elements concentration (for example Fe)), even the N:P:K ratio.
- There is no information of the reagents (organic / inorganic acid) used to adjust the initial pH. It is necessary to know if the reagent can modify the content of ash or C of the hydrochar. Information on final pH should be indicated in this section.
- Equation 1: HC instead of HY. Equation used to calculate liquid yield is required.

Results and discussion:

- I do not find information on C content. A minimum C content is essential for the HTC to make sense.
- I recommend including a title in the y axis of the figures instead of a general title.
- Why is the effect of temperature less significant at pH 4.6? The formation of secondary hydrochar in function of temperature and pH should be discussed in the manuscript.
- Table 1 includes the same information as Figure 2 (ash content). The authors should use only one figure or one table to collect the data. Can the authors define “ash fraction in HC”?
- In what sense does the HTC improve the quality of the residue as fertilizer? Author should explain in depth this affirmation.
- The manuscript does not study the P recovery. They evaluate the P fate depending on the operating conditions. Could the hydrochar be used directly as fertilizer? Could it be used as an intermediate to P extractions? Options for real recovery are required.
- Where is the P in the feedstock? Did the authors analyze P species? The precipitation of P along reaction indicates that the reaction time is a key factor in the process.
- The concentration of heavy metals is collected in Table 2 instead of Table 1.
- One of the explanations for the retention of heavy metals in the hydrochar is associated with porous structure of hydrochar, but hydrochar is characterized by low specific surface. Values of specific surface are required to justify the explanation.
- The authors should justify why T=200°C and pH=4.6 are the optimal conditions to produce a hydrochar with the best qualifications for a solid organo-mineral fertilizer in terms of heavy metal concentration.
- The liquors characterization is collected in Table 3 instead of Table 2.
- Information on P and N balance is required (P or N in the dairy waste with respect to P or N in the HC and in the liquor).
- Subindex NO _3_
- Is the liquor useful for fertirrigation? I don’t understand the optimum nitrate concentration in the liquid phase.
- In conclusion the authors should consider P content, yield and even energy consumption to establish optimum operating conditions. Moreover, a strategy of P-recovery should be included in the manuscript.

I have included some current literature on topic.

Is the study design appropriate and does the work have academic merit?

Partly

Is the work clearly and accurately presented and does it cite the current literature?

Partly

If applicable, is the statistical analysis and its interpretation appropriate?

Partly

Are all the source data underlying the results available to ensure full reproducibility?

Yes

Are the conclusions drawn adequately supported by the results?

Yes

Are sufficient details of methods and analysis provided to allow replication by others?

Partly

Reviewer Expertise:

1. Environmental technologies focused on the wastewatertreatment by means of advanced catalytic and biological processes and wasterecovery; 2. Preparation and application of carbonmaterials from biomass wastes for water treatment and in waste-to-energyconversion and biomass pretreatment for bioenergy; 3. Hydrothermal carbonization of biomass wastes coupling to anaerobic digestion to carry out the valorization of these wastes from a material (synthesis of biocarbon, soil improvers, nutrient recovery, synthesis- of fertilizers) and energetic (solid biofuel, biogas, biohydrogen) point of view.

I confirm that I have read this submission and believe that I have an appropriate level of expertise to confirm that it is of an acceptable scientific standard, however I have significant reservations, as outlined above.

## References

[1] : Phosphorus recovery from aqueous product of hydrothermal carbonization of cow manure. *Waste Manag* .2023;168: 10.1016/j.wasman.2023.06.013 301-310 10.1016/j.wasman.2023.06.013 37331265

[2] : Phosphorus release from hydrothermally carbonized digested sewage sludge using organic acids. *Waste Manag* .2022;151: 10.1016/j.wasman.2022.07.023 60-69 10.1016/j.wasman.2022.07.023 35926282

[3] : Acid-mediated hydrothermal treatment of sewage sludge for nutrient recovery. *Sci Total Environ* .2022;838(Pt 4) : 10.1016/j.scitotenv.2022.156494 156494 10.1016/j.scitotenv.2022.156494 35667432

[4] : Fate of nutrients during hydrothermal treatment of food waste. *Bioresour Technol* .2021;342: 10.1016/j.biortech.2021.125954 125954 10.1016/j.biortech.2021.125954 34592622

# Reviewer report: Reviewer response for version 2

**DOI:** 10.21956/openreseurope.16166.r30264

**Published:** 2022-10-27

**Authors:** Rafal Marcin Lukasik

**Affiliations:** 1 Łukasiewicz Center, Warsaw, Mazowieckie, Poland

Khalaf et al. presented work on the use of dairy wastes in the hydrothermal carbonization process. This work is very interesting contribution to the important area such as the valorisation of complex and not easy wastes. On the other hand, the submitted work demonstrates different than commonly used methods of such kind of wastes valorisation approach. From this perspective, this work can be accepted for indexing.

 However, before this can happen the authors should perform some changes in their work and the most relevant aspects are listed below:

1. In the introduction section one would like to see a short information about the current approaches in dealing with dairy wastes.
2. The results and discussion section authors provide information that achieving the optimal conditions is crucial for the quality of chair as fertilizer. It is rather bizarre to provide the optimisation results at the beginning instead of showing the approach to get it. One would expect that effect of different variables will be discussed and only after that, the optimal conditions will be given as a result.
3. Authors tested different temperature and pH. Why specific range of conditions was selected?
4. Why authors did not use the design of experiments, e.g. Box-Behnken to model the importance of each independent variable? Later, authors could use some polynomial function to better optimise the conditions of HTC process. Even considering various expected outputs, desirability functions could be used to optimise simultaneously several outputs at once as it is given in the literature. ^1^
5. The discussion section should be enriched in the comparison regarding the pros and cons of the used approach vs. other processes of dairy waste valorisation.

After these corrections, the submitted manuscript can be indexed.

Is the study design appropriate and does the work have academic merit?

Partly

Is the work clearly and accurately presented and does it cite the current literature?

Yes

If applicable, is the statistical analysis and its interpretation appropriate?

No

Are all the source data underlying the results available to ensure full reproducibility?

Yes

Are the conclusions drawn adequately supported by the results?

Yes

Are sufficient details of methods and analysis provided to allow replication by others?

Yes

Reviewer Expertise:

Chemical Engineering

I confirm that I have read this submission and believe that I have an appropriate level of expertise to confirm that it is of an acceptable scientific standard, however I have significant reservations, as outlined above.

## References

[1] : Sustainable approach of high-pressure agave bagasse pretreatment for ethanol production. *Renewable Energy* .2020;155: 10.1016/j.renene.2020.04.055 1347-1354 10.1016/j.renene.2020.04.055
